# Malaria and typhoid fever among patients presenting with febrile illnesses in Ga West Municipality, Ghana

**DOI:** 10.1371/journal.pone.0267528

**Published:** 2023-05-25

**Authors:** Tanko Rufai, Enoch Aninagyei, Kwadwo Owusu Akuffo, Christian Teye-Muno Ayin, Priscillia Nortey, Reginald Quansah, Francis Samuel Cudjoe, Ernest Tei-Maya, Isaiah Osei Duah Junior, Anthony Danso-Appiah

**Affiliations:** 1 Department of Epidemiology and Disease Control, School of Public Health, University of Ghana, Legon, Accra, Ghana; 2 Department of Epidemiology and Biostatistics, Arnold School of Public Health, University of South Carolina, Columbia, South Carolina, United States of America; 3 Department of Biomedical Sciences, School of Basic and Biomedical Sciences, University of Health and Allied Sciences, Ho, Ghana; 4 Department of Optometry and Visual Science, College of Science, Kwame Nkrumah University of Science and Technology, Kumasi, Ghana; 5 School of Biomedical and Allied Health Science, University of Ghana, Korle-Bu, Accra; 6 Department of Population, Family and Reproductive Health, School of Public Health, University of Ghana, Legon, Accra, Ghana; 7 Purdue University Biological Sciences, West-Lafayette, Indiana, United States of America; Jazan University, SAUDI ARABIA

## Abstract

**Background:**

Clinicians in areas where malaria and typhoid fever are co-endemic often treat infected patients irrationally, which may lead to the emergence of drug resistance and extra cost to patients. This study determined the proportion of febrile conditions attributable to either malaria and/or typhoid fever and the susceptibility patterns of *Salmonella* spp. isolates to commonly used antimicrobial agents in Ghana.

**Methods:**

One hundred and fifty-seven (157) febrile patients attending the Ga West Municipal Hospital, Ghana, from February to May 2017 were sampled. Blood samples were collected for cultivation of pathogenic bacteria and the susceptibility of the *Salmonella* isolates to antimicrobial agents was performed using the Kirby-Bauer disk diffusion method with antibiotic discs on Müller Hinton agar plates. For each sample, conventional Widal test for the detection of *Salmonella* spp was done as well as blood film preparation for detection of *Plasmodium* spp. Data on the socio-demographic and clinical characteristics of the study participants were collected using an android technology software kobo-collect by interview.

**Results:**

Of the total number of patients aged 2–37 years (median age = 6 years, IQR 3–11), 82 (52.2%) were females. The proportion of febrile patients with falciparum malaria was 57/157 (36.3%), while *Salmonella typhi* O and H antigens were detected in 23/157 (14.6%) of the samples. The detection rate of *Salmonella* spp in febrile patients was 10/157 (6.4%). Malaria and typhoid fever coinfection using Widal test and blood culture was 9 (5.7%) and 3 (1.9%), respectively. The isolates were highly susceptible to cefotaxime, ceftriaxone, ciprofloxacin, and amikacin but resistant to ampicillin, tetracycline, co-trimoxazole, gentamicin, cefuroxime, chloramphenicol, and meropenem.

**Conclusion:**

*Plasmodium falciparum* and *Salmonella spp* coinfections were only up to 1.9%, while malaria and typhoid fever, individually, were responsible for 36.3% and 6.4%, respectively. Treatment of febrile conditions must be based on laboratory findings in order not to expose patients to unnecessary side effects of antibiotics and reduce the emergence and spread of drug resistance against antibiotics.

## Background

Malaria and typhoid fever are two febrile illnesses that cause varying degrees of illness and death in the tropics and sub-tropics, where malaria and typhoid fever are co-endemic. According to the World Health Organization (WHO) global data on malaria published in 2021, about 241 million cases of malaria were diagnosed, with 627, 000 attributable deaths [1]. Two-thirds of these deaths occurred in children under five mostly from Africa [1,2]. At the same time, typhoid and paratyphoid fever, a systemic protracted febrile illness commonly caused by *Salmonella typhi* and common in malaria-endemic settings [3] resulted in 11 to 20 million cases and 20,000 deaths [4]. Due to the lack of rapid, sensitive, and reliable differential diagnosis for *Salmonella* in these settings with mostly weak peripheral health systems and resource constraints, cases of acute febrile illnesses are usually treated presumptively as malaria by clinicians [5]. However, clinical diagnosis is problematic as most malaria-endemic areas are also co-endemic for other infectious diseases that cause fever, such as typhoid fever, urinary tract infection, and pneumonia [6]. Worryingly, diagnosis based on clinical presentation alone is neither sensitive nor specific, resulting in over-treatment in suspected patients [7] thereby developing drug resistance.

The predisposition to malaria and typhoid fever coinfection is usually influenced by similar socio-epidemiological factors and dynamics such as dense populations with poor hygiene and sanitation practices [8,9]. In Ghana, both malaria and typhoid fever were ranked among the twenty leading causes of outpatient morbidities in 2013 [10]. Febrile patients reporting to the Municipal Hospital are usually tested for malaria using the antigen-based point of care- rapid diagnostic test (POC-RDT) at the outpatient department. Similar to practices identified in other studies where malaria POC-RDT results were negative, still, patients were prescribed antimalarials with the potential consequences of over-medicalization [11].

Malaria POC-RDT lacks sensitivity at low parasitemia levels below 50 parasite/μl of blood [12]. Only in severe clinical febrile illness are patients requested to do a Widal test. However, the Widal test lacks specificity compared to culture [13], although blood microscopy (for malaria) and blood, stool, or bone marrow culture (for typhoid fever) remain the definitive diagnostics, they are usually not available in most weak health facilities. Performing separate tests with the definitive diagnostic methods for malaria and typhoid fever on an individual presenting with fever to ascertain true coinfection, to be followed by appropriate treatment, should remain the best option [13] if irrational use of antimalarials and antibiotics, emergence of drug resistance, unnecessary cost and exposure of patients to unnecessary side effects is to be avoided. This study was conducted to provide epidemiological data on coinfection of malaria and typhoid fever using standard diagnostic methods and determine the susceptibility patterns of *Salmonella* isolates to commonly used antimicrobial agents in patients attending the municipal health facility.

## Methods

### Ethical considerations

The Ghana Health Service Ethics Review Committee approved this study (GHS-ERC 52/12/16). Consent was obtained from each patient or guardian of patients aged less than 18 years. Privacy and confidentiality of the study participants were ensured by conducting the interviews in a private and conducive environment.

### Study area and population

The study was conducted at the Ga West Municipal Hospital (GWM) in Amasaman, Accra. The GWM is one of the 29 districts in the Greater Accra Region. The district is 60% rural and 40% peri-urban and urban. The municipality was carved out of the erstwhile Ga District, which was created in 1988 in pursuance of the government’s decentralization and local government reform policy [14]. In 2004, the Ga District was divided into two districts, Ga East, and Ga West, then in 2008 the Ga West District was divided into two, creating Ga West and Ga South Municipalities. In 2018 it was further divided into Ga West and Ga North Municipalities, with Amasaman, remaining the capital of the Ga West Municipal. It lies between latitude 5048’ North and 5039’ North and longitude 0012’ West and 0022’ West. The Municipality shares borders with Ga East and Ga North Municipal Assembly to the southeast, Nsawam Adoagyiri and Akwapem North to the north, and Ga Central and Ga South to the west. The location of this district is a major potential for investment, and it is about 25 km west of Accra, the national capital. It occupies a land area of approximately 145.4 sq km with about 72 communities. The vast tract of land is being developed for both agriculture and estate development The population of the Municipality for 2020 was 127,841 [15]. The municipality is divided into four (4) sub-municipal areas for the purpose of planning and delivery of services, namely: Amasaman, Mayera, Oduman, and Kotoku. The municipality has a district hospital, four health centres, three clinics, four community-based health planning services (CHPS) zone compounds, nine urban CHPS, ten private hospitals and four maternity homes. The Ga West Municipal Hospital is the nearest referral hospital for the smaller health facilities in both Amasaman municipality and its environs, and in turn refers difficult cases to tertiary facilities in Accra.

### Sample preparation and collection

The study sample comprised febrile patients aged 2 years and above. Samples were collected from February to May, 2017 at the outpatients’ department (OPD) in Ga West Municipal Hospital, Amasaman in the Greater Accra region of Ghana. Participation in the study was voluntary and those who refused to take part in the study were still given appropriate attention by the health personnel without any bias. Patients with fever > 37.5°C (temperature taken by an infrared thermometer) presenting to the health facility at the OPD and having consent were included in the study. Patients who met the above criteria but were found to be on antibiotics or antimalarial therapy and those in critical conditions such as convulsion were excluded. Ten (10) milliliters of venous blood were collected aseptically from patients over the age of ten. Out of this volume of blood, 7 mL was inoculated immediately into 45 mL of brain heart infusion (BHI) broth and the remaining 3 mL was transferred into a sterile EDTA tube for malaria diagnosis and typhoid fever serodiagnosis. Similarly, 3–4 mL of blood was collected from children under 10 years old, and 1.5–2 mL of blood was inoculated into 9 mL of BHI broth to isolate *Salmonella* typhi and other *Salmonella* species [16].

### Laboratory procedures

#### Malaria parasites detection

Malaria POC-RDT was done with the SD Bioline rapid diagnostic test kit (Gyeonggi-do, Republic of Korea). This kit determines both *Plasmodium falciparum* histidine rich protein II (*PfhrpII*) and *Plasmodium* lactate dehydrogenase (pLDH) antigens. Also, standard size thick and thin blood films were prepared, thoroughly air-dried and the thin films fixed with absolute methanol for malaria species identification. The blood films were stained with 10% (v/v) Giemsa solution for 20 minutes for the detection of *Plasmodium* parasites and speciation. Malaria microscopy and RDT were done by medical laboratory scientists certified by Ghana Health Service through the On-site Training and Supportive Supervision (OTSS) training program.

#### The traditional Widal test

The Widal agglutination test (Maxwin Global Pharma, Chennai, 600090, India) was performed on all blood samples using the rapid slide titration method to detect *Salmonella* antigens; somatic (O) and flagella (H) antigens (Medsource Ozone Biomedicals, India). Briefly, 50 μL of test serum was placed in two circles on a glass slide and equal volumes each of positive control and normal saline in each of the last two circles respectively. A drop each of O and H antigens was added to the test serum in each circle and then to the negative and positive controls. The content of each circle was mixed using the disposal mixing sticks provided and spread to the entire circle, after which it was rocked gently for 1 minute and observed for agglutination. Antibody titration was performed for the slide reactive samples using the tube technique. According to the manufacturer’s instructions, an antibody titer of > 1: 80 was considered to be significant and usually suggestive of infection, according to the manufacturer.

#### Cultivation, isolation, and identification of *Salmonella* spp from blood

Blood in BHI broth was incubated aerobically at 35–37 °C for 7 days with intermittent cultivation on solid media plates, namely, MacConkey agar (MAC), blood agar (BA) and chocolate agar (CA) on day 2, day 4, and day 6. In the case where BHI broth showed no growth up to day 6, sub-cultures were repeated from the broth on day 7 before it was discarded. Also, on each day of sub-cultivation, broth tubes were examined for turbidity of blood-broth mixture, growth of micro-colonies, haemolysis, colour changes and gas production. For 24–48 hours, the BA and MAC agars were incubated aerobically at 35–37 °C and anaerobically at CA in micro-aerophilic conditions (5–10% CO_2_) for 24–48 hours. This protocol was adapted from a previous study [17]. All isolates from the subcultures were Gram stained and identified through a series of biochemical tests.

#### Biochemical characterization of bacteria

Biochemical tests such as hydrogen sulphide production, presence of oxidase, indole production, carbohydrate metabolism (triple sugar iron agar), citrate utilization and urease production were used to identify pathogens in positive cultures. A sterilized vertical wire loop was used to pick about two colonies and inoculated into the various biochemical tests (triple sugar iron agar, citrate and urea slants). Gram-negative rods that were oxidase negative with the characteristic red slope/yellow butt reaction on triple sugar iron agar either with or without production of H_2_S were confirmed as *Salmonella* species. *S*. *paratyphi* A was differentiated from *S*. *typhi* spp by demonstration weak H_2_S by *S*. *typhi* and no H_2_S production by *S*. *paratyphi* A with gas production by *S*. *paratyphi* A and no gas production by *S*. *typhi*. This protocol has previously been published [18].

#### Antimicrobial susceptibility testing (AST)

The susceptibility of the *Salmonella* isolates to antimicrobial agents was performed using the Kirby-Bauer disk diffusion method with antibiotic discs on Müller Hinton agar plates *(Bauer et al*., 1966). The antibiotics that were used include: Ampicillin (10μg), Co-trimoxazole (25 μg), Tetracycline (10μg), Chloramphenicol (10μg), Ciprofloxacin (5μg), Amikacin (30ug), Gentamicin (10μg), Meropenem (10μg), Cefuroxime (30μg), Ceftriaxone (30μg) and Cefotaxime. About 2–3 colonies of the same morphological type were picked and emulsified in a test tube containing 2.5 mL of sterile peptone water to form a bacterial suspension (equivalent to the density of 0.5 MacFarland standard). A sterile swab stick was dipped into the suspension and uniformly spread on the surface of the Muller-Hinton agar by streaking the entire agar surface [19]. Antibiotic discs obtained from Biomark Laboratory in India were firmly placed on the inoculated medium, inverted, and incubated for 24 hours at 35–37°C. Zones of inhibition were measured after the incubation period. The results were compared with the zone diameter interpretive standards of the Clinical Laboratory Standards Institute NCCLS, 2006 [19].

#### Socio-demographic and clinical data collection

After taking the body’s temperature, those whose temperature reading was ≥37.5°C and had consented were randomly selected. A questionnaire designed and deployed using an android technology, ordinary data kit (ODK) was administered to all participants in the study in English or the two main local languages (Twi or Ga) for those who could not speak English. Each subject was assigned an identification number. The questionnaire was made up of a section on the date of interview and individual serial number; a demographic section including age, sex, marital status, educational level, occupation, and location; and a clinical section covering fever, vomiting, diarrhoea, abdominal pain, joint pain, fatigue, headache, history of transfusion, and antibiotic use; and an environmental section on water source, toilet facility availability, bed net use, hand washing habits, and habit of eating outside home. Randomly selected participants were interviewed, with parents or guardians answering for children aged under 10 years. The questionnaire, which took an average of 15 minutes to administer, was applied by two trained nurses who can communicate in all the three languages.

#### Data management and analysis

Data were double-entered and cleaned in MS Excel before exporting to Stata 13.0 for analysis. Descriptive statistics were presented as percentages. Continuous variables were presented as mean with standard deviation (SD) and median with Interquartile range (IQR), while the Chi square test was used to determine the association of infection status and socio-demographic and clinical variables. Multivariate logistic regression analysis was performed to determine the magnitude and direction of these factors, setting p-value at 0.05 for statistically significant associations.

## Results

### Distribution of febrile malaria and typhoid fever among study participants

Most of the participants positive for febrile malaria were males (54.4%), and aged 18 years and below (89.5%). Most participants had only primary education (75.4%) and majority (82.5%) were employed. Most lived in an urban setting (66.7%), used bed net (66.7%) and did not have a history of travel (87.7%). Similar trends were observed for subjects with typhoid fever. The common source of household water was borehole (80.0%), most infrequently ate outside (90%) and slightly more than two-thirds (70.0%) had a toilet facility as seen in Table 1.

**Table 1:** Distribution of malaria and typhoid fever among the study participants.

Predictors	Febrile malaria				Typhoid fever			
	Positive n (%)	Negative n (%)	χ^2^	*p*-value	Positive n (%)	Negative n (%)	χ^2^	*p*-value
*Sociodemographics*								
Age group (year)			0.078	0.780			0.023	0.881
≤ 18	51 (89.5)	88 (88.0)			9 (90.0)	130 (88.4)		
>18	6 (10.5)	12 (12.0)			1 (10.0)	17 (11.6)		
Gender			1.570	0.210			0.642	0.424
Male	31 (54.4)	44 (44.0)			6 (60.0)	69 (46.9)		
Female	26 (45.6)	56 (56.0)			4 (40.0)	78 (53.1)		
Marital status			0.616	0.735			15.327	0.000
Single	54 (94.7)	93 (93.0)			9 (90.0)	138 (93.9)		
Married	3 (5.3)	6 (6.0)			0 (0.0)	9 (6.1)		
Divorced	0 (0.0)	1 (1.0)			1 (10.0)	0 (0.0)		
Education			1.383	0.710			1.015	0.798
No formal education	10 (17.5)	18 (18.0)			2 (20.0)	26 (17.7)		
Primary	43 (75.4)	76 (76.0)			7 (70.0)	112 (76.2)		
Secondary	2 (3.5)	5 (5.0)			1 (10.0)	6 (4.1)		
Tertiary	2 (3.5)	1 (1.0)			0 (0.0)	3 (2.0)		
Occupation			0.008	0.931			0.059	0.808
Employed	47 (82.5)	83 (83.0)			8 (80.0)	122 (83.0)		
Unemployed	10 (17.5)	17 (17.0)			2 (20.0)	25 (17.0)		
Residence			0.124	0.940			3.614	0.164
Urban	38 (66.7)	65 (65.0)			5 (50.0)	98 (66.7)		
Peri-urban	14 (24.6)	27 (2.0)			5 (50.0)	36 (24.5)		
Rural	5 (8.7)	8 (8.0)			0 (0.0)	13 (8.8)		
*Determinants of febrile malaria*								
Bed net usage			0.045	0.833				
Yes	19(33.3)	35 (35.0)						
No	38(66.7)	65 (65.0)						
History of travel			0.003	0.959			0.626	0.429
Yes	7(12.2)	12 (12.0)			2 (20.0)	17 (11.6)		
No	50(87.7)	88 (88.0)			8 (80.0)	130 (88.4)		
*Determinants of typhoid fever*								
Source of household water							2.174	0.704
Tap water					2 (20.0)	49 (33.3)		
Borehole					8 (80.0)	85 (57.8)		
Packaged water					0 (0.0)	4 (2.7)		
River					0 (0.0)	5 (3.4)		
Others					0 (0.0)	4 (0.0)		
Habit of eating outside home							0.082	0.590
Never					1 (10.0)	10 (6.8)		
Infrequent					9 (90.0)	124 (84.4)		
Frequent					0 (0.0)	13 (8.8)		
Availability of toilet facility							0.820	0.365
Yes					7 (70.0)	120 (81.6)		
No					3 (30.0)	27 (18.4)		
Number of persons in household							2.209	0.137
1					5 (50.0)	106 (72.1)		
2					5 (50.0)	41 (27.9)		

n, frequency; %, percentage frequency; χ^2^, chi-square statistics at a significance of p<0.05.

### Proportion of malaria, typhoid fever, and malaria-typhoid fever coinfection

The commonest presenting signs and symptoms were fever (100%), headache (68.8%), abdominal pain (45.9%), vomiting (36.9%), anorexia (29.3%), joint pain (20.4%), fatigue (15.9%), diarrhea (15.9%), and with having previously used antibiotic during disease onset (100.0%). Of the 157 febrile patients, the prevalence of malaria as determined by RDT and confirmed by microscopy was 36.3% (57/157). In all the parasites identified, mRDT detected *PfhrpII* antigens. Twenty-three (14.6%) of patients had antibody titers (both O and H antigens) for *Salmonella spp* while 10 (6.4%) were positive for blood culture. Diagnosing typhoid fever with Widal test and blood culture, coinfection with malaria was 9 (5.7%) and 3 (1.9%), respectively.

### Factors associated with malaria and culture positive *S*. *typhi* infections

Table 2 shows logistic regression analysis of the determinants of febrile malaria and typhoid fever. No particular factor significantly predicted risk of infection with malaria or typhoid in this study.

**Table 2:** Factors associated with febrile malaria and typhoid fever infections.

Predictors	Febrile malaria			Typhoid fever		
	COR	95% CI	p-value	COR	95% CI	p-value
Age group (year)						
≤ 18	Ref			Ref		
>18	1.16	0.41–3.28	0.781	1.18	0.14–9.87	0.881
Gender						
Male	Ref			Ref		
Female	1.52	0.78–2.92	0.211	1.7	0.46–6.26	
Marital status						
Single/Divorced	Ref			Ref		
Married	2.01	0.40–10.03	0.394	-	-	0.999
Education						
No formal education	Ref			Ref		
Primary	0.42	0.156–1.11	0.790	1.23	0.24–6.27	0.803
Secondary/Tertiary	0.64	0.13–3.25	0.586	0.69	0.06–8.58	0.775
Occupation						
Employed	Ref			Ref		
Unemployed	0.96	0.41–2.27	0.931	0.82	0.16–4.09	0.809
Residence						
Urban	Ref			Ref		
Peri-urban /Rural	0.56	0.29–1.12	0.98	0.5	0.14–1.81	0.219
Determinants of febrile malaria						
Bed net usage						
Yes	Ref					
No	0.93	0.47–1.85	0.833			
History of travel						
Yes	Ref					
No	1.03	0.38–2.78	0.959			
Determinants of typhoid fever						
Source of household water						
Tap water				Ref		
Borehole				0.43	0.09–2.12	0.303
Habit of eating outside home						
Never				Ref		
Infrequent				1.38	0.16–12.00	0.77
Frequent				-	-	0.999
Availability of toilet facility						
Yes				Ref		
No				0.53	0.13–2.16	0.372
Number of persons in household						
1				Ref		
2				0.39	0.11–1.41	0.149

Bivariate regression analysis at a significance of p < 0.05; C*OR*, crude odds ratio; *CI*, confidence interval; *Ref*, reference.

### Antimicrobial susceptibility profiles of *Salmonellae* isolates

All isolates exhibited resistance against ampicillin, tetracycline, co-trimoxazole, gentamicin, cefuroxime, chloramphenicol and meropenem while the isolates showed susceptibility to cefotaxime, ceftriaxone, ciprofloxacin, and amikacin (Table 3).

**Table 3:** Antimicrobial susceptibility profile of *Salmonella* isolates from typhoid fever.

Antibiotic	*S*. *typhi* N = 10 (%)	*S*. *paratyphi* N = 3 (%)	Other *Salmonella* spp. N = 2 (%)	Total N = 15 (%)
Ampicillin	8 (80) R	3 (100) R	2 (100) R	13 (86.7) R
Tetracycline	9 (90) R	3 (100) R	2 (100) R	14 (93.3) R
Co-trimoxazole	10 (100) R	3 (100) R	2 (100) R	15 (100) R
Gentamicin	10 (100) R	2 (66.7) R	2 (100) R	14 (93.3) R
Cefotaxime	7 (70) S	2 (66.7) S	1 (50) S	10 (66.7) S
Cefuroxime	10 (100) R	3 (100) R	1 (50) R	14 (93.3) R
Chloramphenicol	6 (60) R	1 (33.3) R	2 (100) R	9 (60) R
Ceftriaxone	6 (60) R	2 (66.7) S	1 (50) S	11 (73.3) S
Ciprofloxacin	10 (100) S	3 (100) S	2 (100) S	15 (100) S
Amikacin	10 (100) S	3 (100) S	2 (100) S	15 (100) S
Meropenem	8 (80.0) R	2 (66.7) R	2 (100.0) R	12 (80.0) R

R, resistant; S, susceptible; %, percentage frequency.

## Discussion

Malaria and typhoid fever continue to be major diseases of public health concern, especially in tropical countries, and are known to present clinically similar symptoms, with fever being the major presentation [20]. Our investigation showed that the common clinical complaint reported by patients with suspected malaria or typhoid fever was fever, followed by headache, then abdominal pain. In the current study, 36.3% of patients with febrile illness were found to have malaria and 14.6% and 6.4% had typhoid fever using the traditional Widal test and blood culture, respectively.

Even though the two diseases are endemic in Ghana, findings from this study show that malaria was implicated as the cause of most febrile illnesses. Studies conducted elsewhere in Ghana reported a marginally higher prevalence of malaria (18.6%) than typhoid fever (17%) [21]. However, prevalence of malaria was found to be much higher than typhoid fever in this setting. Comparing widal test to blood culture assay (the gold standard diagnosis for typhoid fever), there was overestimation using the Widal test which gives a false positive of (8.3%) and this could be due to the persistence of *Salmonella* antibodies in the patients [22] or cross-reactivity of *Salmonella* H and O antigens with other pathogens [23].

Typhoid fever prevalence in this study, using the Widal test, is comparable to findings in Northwest Ethiopia (19.0%) [24] and Ibadan, (16.7%) [25] but much lower compared to other studies in Sierra Leone, (31.4%) [26], and Kaduna State, (36.6%) [13]. Differences in prevalence could be due to differences in Widal test kits used, seasonal variations during which the study was carried out, differences in cultural practices especially with respect to handwashing as well as availability of toilet facilities. Additionally, there is a variation in the baseline antibody titres of healthy individuals in different geographical settings, making the interpretations of the results complex [27]. Widal test is also known to cross-react with *Plasmodium* antibodies [28]. Due to these limitations, reason, diagnosis of typhoid fever using blood culture is preferred.

A previous study in Ghana [29], Cameroon [30], Nigeria [3,31], Ethiopia [24] and India [13,32] used blood culture to diagnose typhoid fever in febrile patients. This study found that 49.0% of the fevers were neither malaria nor typhoid. Clearly, other clinical conditions are also associated with fever. A few of them are sickle cell [33], viral infections [34], and other haemo-parasitic infections such as babesiosis [35]. It could also be a result of systemic inflammation [36]. Other bacterial infections such as leptospirosis [37], *Mycoplasma pneumoniae* infection [38], streptococcal throat infection [37] and meningitis [39] are also known to be associated with pyrexia. A study in Tanzania also found patients presenting with fever-related febrile conditions such as Q fever and brucellosis, were undetected in a large number of patient diagnoses, whiles malaria accounted for only 1.6% of all febrile illnesses [40]. These findings underscore the existence of non-malarial and non-typhoid febrile illnesses in both malaria and typhoid fever endemic regions which can only be detected with good diagnostic facilities. Therefore, the need for innovative biomedical technologies to aid diagnosis cannot be overemphasized.

It was found that insecticide-treated bed net usage was not associated with malaria even though about 65% of the patients did not use insecticide-treated bed nets. Insecticide-treated nets have been shown to be highly efficient at reducing malaria incidences [41]. Also, the source of drinking water was not associated with typhoid fever, though such an association has been previously found [42]. The frequency of typhoid fever was higher in study participants who bought food from a vendor. This observation could be due to unhygienic conditions that characterize commercial food preparation [43]. Habit of eating outside home, toilet facilities at home, and sources of water for drinking were also not significantly associated with typhoid fever. This finding is supported by a study in Ethiopia [24].

Malaria and other bacterial coinfection have been previously reported. Pathogens such as *Salmonella enterica* serovar and *Escherichia coli* [44], *Klebsiella pneumonia*, *Rickettsia felis*, *Acinetobacter* spp., *Klebsiella spp*., *Haemophilus influenza*, *Pseudomonas* spp., *Enterobacter cloacae*, *Serratia marcescens*, *Aeromonas* spp and *Campylobacter* spp [45] Surprisingly, the *Salmonella* pathogens isolated in this study were found to be resistant to seven out of the eleven (63.6%) antibiotics tested. The antibiotics found not to be effective against the pathogens were the penicillins (ampicillin), the sulphonamide (co-trimoxazole), tetracycline, the aminoglycoside (gentamicin), cephalosporin (cefuroxime), chloramphenicol (inhibitor of protein synthesis) and the carbapenem (meropenem). However, another penicillin class of antibiotics, amikacin, two cephalosporins that are β-lactam agents (cefotaxime and ceftriaxone) and the only fluoroquinolone (ciprofloxacin) were highly sensitive against the isolated pathogens. As reported in this study, several other studies in Ghana and elsewhere have reported high prevalence of ampicillin, co-trimoxazole, tetracycline, gentamicin, cefuroxime, chloramphenicol, and meropenem resistant blood pathogens [46–49]. The resistance of blood pathogens to these antibiotics in this study could be associated with frequent use of these antibiotics in this setting. In this and other studies, amikacin has been found to be very effective against most bacteria, probably because of its resistance to most aminoglycoside-modifying enzymes. Amikacin has been widely used successfully to treat bacteria that were resistant to other aminoglycosides [50–52]. The cephalosporin class of antibiotics, especially the 3rd generation types, have shown tremendous success in treating Gram-negative infections such as salmonellosis. These cephalosporins are resistant to beta-lactamases produced by some bacteria [53,54]. Multiple Plasmid–Mediated Quinolone Resistance (PMQR) resulting in resistance to ciprofloxacin in *Salmonella* species has increasingly been reported in some studies [55,56]. However, this study together with several others, found ciprofloxacin to be sensitive to *Salmonella* species [57–59]. Ciprofloxacin is known to exert it bactericidal action by inhibiting DNA replication through inhibiting bacterial DNA topoisomerase and DNA-gyrase [60].

Some limitations of this study are the small sample size which might have reduced the statistical power to detect statistical differences. However, given the greater stability and less variability of the assessed parameters the sample size remains adequate and unlikely to influence the conclusion of the study. Secondly, only the standard manual method (blood culture) was used in isolating the organisms and this could have been done alongside molecular techniques involving PCR which are more sensitive and would have been more accurate. Thirdly, serotyping for antigenic structure and grouping of *Salmonellae* species was not done.

## Conclusion

Malaria and typhoid comorbidity were less than 2% of the total samples analyzed. Individually, more than five times malaria fever were presented compared to typhoid fever. Due to the low prevalence of malaria and typhoid fever coinfection, clinicians should not treat concurrently but rather stick to differential diagnosis to ensure judicious use of resources.

## Implications of these findings

The implications of these findings are enormous. Based on these findings and reports from other studies, malaria was the cause of fever among febrile patients rather than typhoid fever. Meanwhile, the prevalence of malaria and typhoid fever coinfection was also found to be low. Therefore, treating bacterial infections without previous determination of antibiogram does not help the control of bacterial infections and also leads to the creation of drug-resistant strains. It is recommended that all bacterial and parasitic infections be treated by clinicians based on a laboratory diagnosis and antibiogram profile. This is because there is a chance that most commonly available antibiotics or antimalarials may not be sensitive to the pathogen causing the infection. Again, to reduce the risk of antimicrobial resistance development and the spread of resistant bacteria, healthcare organizations like Ghana Health Service must enact or re-enforce procedures to ensure the optimal use of antibiotics. This can be achieved by implementing antimicrobial stewardship programs. Organizations can provide appropriate antibiotics to patients requiring antibiotic treatment. Additionally, these programs ensure the correct antibiotic is prescribed at the right time, dose, and duration. Collectively, these measures improve patient health outcomes and preserve the effectiveness of antimicrobial treatments.

## References

[1] WHO. World malaria report 2020: 20 years of global progress and challenges. World Health Organization. 2020.

[2] Mbacham W, Ayong L, Guewo-Fokeng M, Makoge V. Current situation of malaria in Africa. Malaria Control and Elimination: Springer; 2019. p. 29–44. 10.1007/978-1-4939-9550-9_2 31267491

[3] Nwuzo A, Onyeagba R, Iroha I, Nworie O, Oji A. Parasitological, bacteriological, and cultural determination of prevalence of malaria parasite (Plasmodium falciparum) and typhoid fever co-infection in Abakaliki, Ebonyi State. Scientific Research Essays. 2009;4(10):966–71.

[4] Bonville C, Domachowske J. Adenovirus. Vaccines: Springer; 2021. p. 89–97.

[5] Stoler J, Awandare GA. Febrile illness diagnostics and the malaria-industrial complex: a socio-environmental perspective. BMC Infect Dis. 2016;16(1):683. doi: 10.1186/s12879-016-2025-x 27855644 PMC5114833

[6] Chandramohan D, Jaffar S, Greenwood B. Use of clinical algorithms for diagnosing malaria 1. Tropical Medicine International Health. 2002;7(1):45–52. 11851954 10.1046/j.1365-3156.2002.00827.x

[7] Reyburn H, Ruanda J, Mwerinde O, Drakeley C. The contribution of microscopy to targeting antimalarial treatment in a low transmission area of Tanzania. Malaria Journal 2006;5(1):1–6. 16423307 10.1186/1475-2875-5-4 PMC1360087

[8] Iheukwumere I, Nwachukwu CN, Kanu MA. Manifestations, mismanagement and diagnostic challenges of malaria and typhoid fever. Malar Chemoth Cont Elimination. 2013;2(109):38–41.

[9] Sharma B, Matlani M, Gaind R, Pandey K. Malaria and typhoid co-infection in India: a diagnostic Difficulty. Journal of Dental Medical Sciences. 2016;15(9):101–04.

[10] GHS. THE HEALTH SECTOR IN GHANA FACTS AND FIGURES 2015. GHANA HEALTH SERVICE. 2015.

[11] Manyando C, Njunju EM, Chileshe J, Siziya S, Shiff C. Rapid diagnostic tests for malaria and health workers’ adherence to test results at health facilities in Zambia. Malaria Journal 2014;13(1):1–8. doi: 10.1186/1475-2875-13-166 24885996 PMC4026818

[12] Aninagyei E. Repeated sampling improved the sensitivity of malaria microscopy in children under six years. BMC Res Notes. 2020;13(1):508. doi: 10.1186/s13104-020-05359-w 33148309 PMC7640445

[13] Mbuh FA, Galadima M, Ogbadu L. Rate of co-infection with malaria parasites and Salmonella Typhi in Zaria, Kaduna State, Nigeria. Annals of African Medicine. 2003;2(2):64–7.

[14] GWMA. Ga West Municipal Asembly (2021). 2021.

[15] GSS. 2010 Population and housing census. GSS (Ghana Statistical Service) (2010). 2010.

[16] Cattoir L, Claessens J, Cartuyvels R, Van den Abeele A. How to achieve accurate blood culture volumes: the BD BACTEC FX blood volume monitoring system as a measuring instrument and educational tool. European Journal of Clinical Microbiology & Infectious Diseases. 2018;37(9):1621–6. doi: 10.1007/s10096-018-3291-x 29882176

[17] Opota O, Croxatto A, Prod’hom G, Greub G. Blood culture-based diagnosis of bacteraemia: state of the art. Clinical Microbiology and Infection. 2015;21(4):313–22. doi: 10.1016/j.cmi.2015.01.003 25753137

[18] Monica C. District laboratory practice in tropical countries. Cambridge University Press; 2006.

[19] Wikler MA. Performance standards for antimicrobial susceptibility testing Sixteenth informational supplement. M 100-S 16. 2006.

[20] Igharo EA, Osazuwa F, Ajayi SA, Ebueku A, Igbinigie O. Dual infection with typhoid and malaria in febrile patients in Ikare Akoko, Nigeria. International Journal of Tropical Medicine. 2012;7(1):49–52.

[21] Afoakwah R, Acheampong D, Boampong J, Sarpong-Baidoo M, Nwaefuna E, Tefe P. Typhoid-Malaria co-infection in Ghana. Exp Biol. 2011;1(3):1–6.

[22] House D, Wain J, Ho VA, Diep TS, Chinh NT, Bay PV, et al. Serology of typhoid fever in an area of endemicity and its relevance to diagnosis. J Clin Microbiol. 2001;39(3):1002–7. doi: 10.1128/JCM.39.3.1002-1007.2001 11230418 PMC87864

[23] Purwaningsih S, Handojo I, Prihatini, Probohoesodo Y. Diagnostic value of dot-enzyme-immunoassay test to detect outer membrane protein antigen in sera of patients with typhoid fever. Southeast Asian J Trop Med Public Health. 2001;32(3):507–12. 11944708

[24] Birhanie M, Tessema B, Ferede G, Endris M, Enawgaw B. Malaria, typhoid fever, and their coinfection among febrile patients at a rural health center in Northwest Ethiopia: a cross-sectional study. Advances in medicine. 2014;2014. 10.1155/2014/531074 PMC4590977 26556415

[25] Igbeneghu C, Olisekodiaka M, Onuegbu J. Malaria and typhoid fever among adult patients presenting with fever in Ibadan, Southwest Nigeria. International journal of tropical medicine. 2009;4(3):112–5.

[26] Sundufu A, James M, Foday I. Role of co-infection with malaria parasites and Salmonella Typhoid in Bo City. Southern Sierra Leone Public Health Research. 2012;2(6):204–7.

[27] Kumari CA, Rajasekhar K, Kirani KS, Murthy DS. Study of Baseline Titres for Widal test among Population in Kakinada Region of AP.

[28] Pradhan P. Coinfection of typhoid and malaria. Journal of medical laboratory diagnosis. 2011;2(3):22–6.

[29] Labi AK, Obeng-Nkrumah N, Addison NO, Donkor ES. Salmonella blood stream infections in a tertiary care setting in Ghana. BMC Infect Dis. 2014;14:3857. doi: 10.1186/s12879-014-0697-7 25528352 PMC4297363

[30] Ndip LM, Egbe FN, Kimbi HK, Njom HA, Ndip RN. Co-infection of malaria and typhoid fever in feverish patients in the Kumba health district, Southwest Cameroon: public health implications. J IJTDH. 2015;9(4):1–11.

[31] Orok DA, Usang AI, Ikpan OO, Duke EE, Eyo EE, Edadi UE, et al. Prevalence of malaria and typhoid fever co-infection among febrile patients attending college of health technology medical centre in Calabar, Cross River state, Nigeria. 2016.

[32] Samatha P, Rao CK, Sowmya SB. Malaria typhoid co-infection among febrile patients. Journal of Evolution of Medical Dental Sciences. 2015;4(65):11322–8.

[33] Wierenga KJ, Hambleton IR, Wilson RM, Alexander H, Serjeant BE, Serjeant GR. Significance of fever in Jamaican patients with homozygous sickle cell disease. Arch Dis Child. 2001;84(2):156–9. doi: 10.1136/adc.84.2.156 11159294 PMC1718651

[34] Koskiniemi M-L, Vaheri A. Acute encephalitis of viral origin. Scandinavian Journal of Infectious Diseases. 1982;14(3):181–7. doi: 10.3109/inf.1982.14.issue-3.05 6293046

[35] Hatcher JC, Greenberg PD, Antique J, Jimenez-Lucho VE. Severe babesiosis in Long Island: review of 34 cases and their complications. Clinical infectious diseases. 2001;32(8):1117–25. doi: 10.1086/319742 11283800

[36] Evans SS, Repasky EA, Fisher DT. Fever and the thermal regulation of immunity: the immune system feels the heat. Nat Rev Immunol. 2015;15(6):335–49. doi: 10.1038/nri3843 25976513 PMC4786079

[37] El-Radhi AS. Fever in common infectious diseases. Clinical Manual of Fever in Children: Springer; 2018. p. 85–140.

[38] Mohammed H, Hassan M, Bakir S. Campylobacter jejuni gastroenteritis in children in Basrah-Iraq. Mjbu. 2004;22(1&2):1–5.

[39] Wong V, Hitchcock W, Mason W. Meningococcal infections in children: a review of 100 cases. The Pediatric infectious disease journal. 1989;8(4):224–7. 2654860

[40] Crump JA, Morrissey AB, Nicholson WL, Massung RF, Stoddard RA, Galloway RL, et al. Etiology of severe non-malaria febrile illness in Northern Tanzania: a prospective cohort study. PLoS neglected tropical diseases. 2013;7(7):e2324. doi: 10.1371/journal.pntd.0002324 23875053 PMC3715424

[41] Klinkenberg E, Onwona-Agyeman KA, McCall PJ, Wilson MD, Bates I, Verhoeff FH, et al. Cohort trial reveals community impact of insecticide-treated nets on malariometric indices in urban Ghana. Trans R Soc Trop Med Hyg. 2010;104(7):496–503. doi: 10.1016/j.trstmh.2010.03.004 20417945

[42] Achonduh-Atijegbe OA, Mfuh KO, Mbange AH, Chedjou JP, Taylor DW, Nerurkar VR, et al. Prevalence of malaria, typhoid, toxoplasmosis and rubella among febrile children in Cameroon. BMC infectious diseases. 2016;16(1):1–9. doi: 10.1186/s12879-016-1996-y 27825318 PMC5101675

[43] Parry CM. Epidemiological and clinical aspects of human typhoid fever. Salmonella infections: clinical, immunological molecular aspects. 2006:1–24.

[44] Sandlund J, Naucler P, Dashti S, Shokri A, Eriksson S, Hjertqvist M, et al. Bacterial coinfections in travelers with malaria: rationale for antibiotic therapy. Journal of Clinical Microbiology. 2013;51(1):15–21. doi: 10.1128/JCM.02149-12 23052321 PMC3536217

[45] Wilairatana P, Mala W, Masangkay FR, Kotepui KU, Kotepui M. The Prevalence of Malaria and Bacteremia Co-Infections among Febrile Patients: A Systematic Review and Meta-Analysis. Tropical medicine and infectious disease. 2022;7(9):243. doi: 10.3390/tropicalmed7090243 36136654 PMC9503679

[46] Adzitey F. Antibiotic resistance of Escherichia coli and Salmonella enterica isolated from cabbage and lettuce samples in Tamale metropolis of Ghana. International Journal of Food Contamination. 2018;5(1):1–7.

[47] Kwaku GM, Samson SP, Charles M-RF. Resistance of bacteria isolates from cabbage (Brassica oleracea), carrots (Daucus carota) and lettuce (Lactuca sativa) in the Kumasi Metropolis of Ghana. Int J Nutr Food Sci 2016;5(4):297–303.

[48] Firoozeh F, Shahcheraghi F, Salehi TZ, Karimi V, Aslani M. Antimicrobial resistance profile and presence of class I integrongs among Salmonella enterica serovars isolated from human clinical specimens in Tehran, Iran. Iranian journal of microbiology. 2011;3(3):112. 22347592 PMC3279819

[49] Ahmed AM, Younis EE, Ishida Y, Shimamoto T. Genetic basis of multidrug resistance in Salmonella enterica serovars Enteritidis and Typhimurium isolated from diarrheic calves in Egypt. Acta tropica. 2009;111(2):144–9. doi: 10.1016/j.actatropica.2009.04.004 19375408

[50] Gerding DN, Larson TA, Hughes RA, Weiler M, Shanholtzer C, Peterson LR. Aminoglycoside resistance and aminoglycoside usage: ten years of experience in one hospital. Antimicrob Agents Chemother. 1991;35(7):1284–90. doi: 10.1128/AAC.35.7.1284 1929283 PMC245159

[51] Gad GF, Mohamed HA, Ashour HM. Aminoglycoside resistance rates, phenotypes, and mechanisms of Gram-negative bacteria from infected patients in upper Egypt. PLoS One. 2011;6(2):e17224. doi: 10.1371/journal.pone.0017224 21359143 PMC3040769

[52] Marsot A, Guilhaumou R, Riff C, Blin O. Amikacin in Critically Ill Patients: A Review of Population Pharmacokinetic Studies. Clin Pharmacokinet. 2017;56(2):127–38. doi: 10.1007/s40262-016-0428-x 27324191

[53] Richards D, Heel R, Brogden R, Speight T, Avery G. Ceftriaxone. Drugs 1984;27(6):469–527. 6329638 10.2165/00003495-198427060-00001

[54] Jones RN, Thornsberry C. Cefotaxime: a review of in vitro antimicrobial properties and spectrum of activity. Rev Infect Dis. 1982;4 Suppl:S300–15. doi: 10.1093/clinids/4.supplement_2.s300 6294779

[55] Cui S, Li J, Sun Z, Hu C, Jin S, Guo Y, et al. Ciprofloxacin-resistant Salmonella enterica serotype Typhimurium, China. Emerg Infect Dis. 2008;14(3):493–5. doi: 10.3201/eid1403.070857 18325271 PMC2570801

[56] Threlfall EJ, Ward LR. Decreased susceptibility to ciprofloxacin in Salmonella enterica serotype typhi, United Kingdom. Emerg Infect Dis. 2001;7(3):448–50. doi: 10.3201/eid0703.010315 11384525 PMC2631792

[57] Khanal B, Sharma SK, Bhattacharya SK, Bhattarai NR, Deb M, Kanungo R. Antimicrobial susceptibility patterns of Salmonella enterica serotype typhi in eastern Nepal. J Health Popul Nutr. 2007;25(1):82–7. 17615907 PMC3013267

[58] Davis R, Markham A, Balfour JA. Ciprofloxacin. An updated review of its pharmacology, therapeutic efficacy and tolerability. Drugs. 1996;51(6):1019–74. doi: 10.2165/00003495-199651060-00010 8736621

[59] C-R DM, Monk J, Price A, Benfield P, Todd P, WARD A. Ciprofloxacin. A review of its antibacterial activity, pharmacokinetic properties and therapeutic use. Drugs. 1988;35(4):373–447. doi: 10.2165/00003495-198835040-00003 3292209

[60] Fisher LM, Lawrence JM, Josty IC, Hopewell R, Margerrison EE, Cullen ME. Ciprofloxacin and the fluoroquinolones: new concepts on the mechanism of action and resistance. The American journal of medicine. 1989;87(5):S2–S8. 10.1016/0002-9343(89)90010-7 2574005

